# Burnout, anxiety and depression risk in medical doctors working in KwaZulu-Natal Province, South Africa: Evidence from a multi-site study of resource-constrained government hospitals in a generalised HIV epidemic setting

**DOI:** 10.1371/journal.pone.0239753

**Published:** 2020-10-14

**Authors:** Thejini Naidoo, Andrew Tomita, Saeeda Paruk

**Affiliations:** 1 Postgraduate Programme (Master of Medicine), Discipline of Psychiatry, School of Clinical Medicine, College of Health Sciences, University of KwaZulu-Natal, Durban, South Africa; 2 KwaZulu-Natal Research Innovation and Sequencing Platform (KRISP), College of Health Sciences, University of KwaZulu-Natal, Durban, South Africa; 3 Centre for Rural Health, School of Nursing and Public Health, College of Health Sciences, University of KwaZulu-Natal, Durban, South Africa; 4 Discipline of Psychiatry, School of Clinical Medicine, College of Health Sciences, University of KwaZulu-Natal, Durban, South Africa; University of Oxford, UNITED KINGDOM

## Abstract

Globally, burnout in medical doctors (MDs) is concerning, with higher rates reported in studies conducted in South Africa (SA). This psychological syndrome leads to serious health consequences, and jeopardises patient care. Despite this, there is no data pertaining to these potential adverse mental health outcomes in KwaZulu-Natal (KZN) Province, SA, where it is overshadowed by the fight against priorities such as HIV and AIDS/TB. This study therefore aimed to establish the nature and extent of burnout, anxiety and depressive symptoms and their associations among public sector MDs in KZN. A cross sectional study was conducted among MDs at five KZN public sector training hospitals to investigate their associations with practitioner (individual) and organisational factors using the Maslach Burnout Inventory–Human Services Survey (MBI-HSS), the Generalised Anxiety Disorder-7 (GAD-7) questionnaire and the Patient Health Questionnaire-9 (PHQ-9). Of the 150 participants, 88 (59.0%) screened positive for burnout, as indicated by high scores on the emotional exhaustion or depersonalisation subscales in the MBI-HSS. One fifth screened positive for anxiety (n = 30) and depressive symptoms (n = 32). Burnout was significantly associated with individual factors of anxiety (p<0.01) and depressive (p<0.01) symptoms based on adjusted logistic regression models. Organisational factors, such as lack of clinical supervisor support (p<0.01) and hospital resources (p<0.01), were significantly associated with burnout based on the bivariate analyses. Burnout, anxiety and depressive symptoms in MDs are highly prevalent and intertwined in resource constrained KZN public training hospitals. Addressing burnout at individual and organisational levels is important to mitigate its adverse effects.

## Introduction

The dehumanisation of modern medicine, high burden of patients, and lack of adequate resources affects not only patients but medical doctors (MDs). It manifests itself in multiple adverse mental health outcomes, such as burnout, anxiety and depression [1]. Maslach and colleagues defined burnout as a multi-dimensional construct that can be conceptualised as a ‘psychological syndrome in response to chronic interpersonal stressors on the job’. Burnout comprises of three components: emotional exhaustion, depersonalisation and personal accomplishment [1]. Emotional exhaustion, the individual stress dimension of burnout, bears reference to both emotional and physical fatigue over an extended period. Depersonalisation, the interpersonal dimension of burnout, refers to harbouring feelings of negativity toward and detachment from the job. Poor personal accomplishment, the self-evaluative dimension of burnout, refers to feelings of incompetence and a lack of achievement at work. While the first two dimensions arise from the presence of work overload and social conflict, the latter arises from a lack of resources [1].

Globally, the prevalence of burnout in MDs is highly variable due to the heterogeneity of burnout assessment methods, definitions and outcomes as well as statistical heterogeneity [2–5]. In addition, despite most studies using the Maslach Burnout Inventory (MBI) to screen for burnout, there were multiple implementations of the MBI versions, cut-off combinations, or both [2]. High rates of burnout were also uncovered by a cross sectional, national survey of medical doctors in South Africa (SA) in 2003 [6]. In the Western Cape (WC) Province, SA, a 2011 cross-sectional study revealed that 100% of junior doctors experienced a high degree of burnout on one of the three subscales on the MBI. In another 2013 WC Province study, burnout was reported to be 76% among MDs, mainly medical officers working in public sector clinics and district hospitals [7,8]. High levels of burnout have also been reported in MDs working in Gauteng Province [9] and Bloemfontein, SA [10].

Individual and organisational (interpersonal and institutional) factors associated with burnout include: work stress and anxiety; balancing work and personal life; long working hours; high workloads; poor working conditions; public system-related frustrations; insufficient vacation time; inadequate equipment; poor management support and low work satisfaction [7,11–15]. Furthermore, the HIV and AIDS epidemic and the mass exodus of MDs from SA places further strain on those remaining in the public sector [7,8]. These factors have a negative impact on patient care, and increase the risk for psychiatric co-morbidities, such as suicide, anxiety and depression [8]. The emotional exhaustion component of burnout has been closely linked to anxiety [16], whereas burnout and depression are considered distinct entities that may have a causal link [17].

The prevalence estimates for anxiety in the global population in 2017 was 3.6% [18] and 8.1% in SA, much lower than that reported in MDs. A study conducted in the United Kingdom (UK), reported that 29% of doctors were experiencing symptoms of anxiety [19], and rates of 14.6% in Turkish emergency doctors have been documented [20]. In a more recent 2016 study in Pakistan, 40% of MDs reported anxiety symptoms [21].

Outside of SA, no studies were found exploring anxiety in medical doctors in Africa. A 2007 study conducted in MDs working in the WC, SA, reported that the combined prevalence of anxiety and depression was 53% [22]. Symptoms of anxiety and depression are commonly co-morbid [23–25], and have been linked to adverse outcomes in social, occupational and physical realms of functioning [26–28].

The prevalence estimates for depression in the global population in 2017 was 4.4% [18], with a 2015 meta-analysis showing that the pooled prevalence of depressive symptoms in MDs was 28.8%, with a range from 20.9% to 43.2% [29]. In a WC (SA) study, depressive symptom rates approximated 30%, in-keeping with global figures [7,19–21,29]. A recent 2019 study in the WC demonstrated that 40.7% of medical interns experienced depressive symptoms [30].

MDs thus represent a high-risk population for burnout, anxiety and depression internationally and in SA. In the SA context, the KwaZulu-Natal (KZN) Provincial Department of Health (DoH) has recently faced many financial and human resource challenges [31]. Increased work demands and a substantive HIV anti-retroviral treatment roll- out program within a very limited resource setting predisposing health care professionals to stress and burnout [31]. KZN has the highest HIV prevalence in the country, and provincial commitments towards mental health services for its MDs may be overshadowed by the realities of prioritising the considerable HIVand AIDS or TB challenges [32].

As stated earlier there are no studies on anxiety in MDs in Africa. In addition, there is a paucity of studies examining these three variables together [33] in MDs internationally [34], and there are no studies of this nature from Africa. The aim of this study (hereafter labelled as the ZABRE study which stands for South African Burnout Epidemiology) was to investigate the prevalence of burnout, anxiety and depressive symptoms and their associations with practitioner (individual) and organisational factors in MDs in KZN public sector training hospitals prior to the global coronavirus pandemic. This study is integral to monitoring burnout trends in different regions over time, and serves to ascertain the most current variables associated with burnout, anxiety and depression so that public mental health interventions can be tailored to the specific needs of MDs in the unique KZN context.

## Methods

### Study design, setting, participants and procedure

This study was a cross-sectional, descriptive, quantitative, self-administered questionnaire survey of MDs. The five recruitment sites were selected due to their being regional academic/training public sector hospitals in the eThekwini Municipality of KZN, SA. The MDs appointed here are responsible for training medical students, interns and registrars (residents). Any MD in full-time employment who was registered as an intern, community service officer or for independent practice with the Health Professional Council of SA (HPCSA), in an academic or non-academic clinical post, who was willing to participate in the study was eligible to be included. Those not willing to participate or who were in part-time employment were excluded. Participants were approached via their departmental academic meetings or in their respective clinical work areas. The data was collected from September 2018 to January 2019 by the PI.

The study was approved by the Biomedical Research Ethics Committee (BREC) of the University of KwaZulu-Natal (UKZN), the KZN DoH and the hospital managers. The BREC reference number is BE013/18. Written informed consent was obtained from all participants prior to their enrolment in the study. A mental health resource help sheet that included support information, such as the contact details for the Department of Psychiatry, support groups and public hospitals with mental health services was provided to MDs irrespective of whether they participated in the study.

### Outcomes

Participants completed four questionnaires:

1. detailing their socio-demographic and occupational profile;
2. the Maslach Burnout Inventory–Human Services Survey to measure burnout;
3. the Generalised Anxiety Disorder-7 questionnaire to measure anxiety; and
4. the Patient Health Questionnaire-9 to screen for depressive symptoms.

The main study outcomes measured were (1) burnout, (2) anxiety and (3) depressive symptoms.

Burnout was assessed using the Maslach Burnout Inventory–Human Services Survey (MBI-HSS), this being the original and most widely used version. The questionnaire is appropriate for use in participants from a wide array of occupations, including MDs. It consists of 22-items answered on a 7-point Likert scale, with responses ranging from ‘never’ to ‘everyday’. Burnout has been positively correlated to high scores in the emotional exhaustion and depersonalisation subscales, and low scores in the personal accomplishment subscale. A score of 27 and above in the emotional exhaustion subscale was considered high; 19–26 moderate; and less than 19 low [35]. A score of 10 and above in the depersonalisation subscale was considered high; 6–9 moderate; and less than 6 low [35]. A score of 40 and above in the personal accomplishment subscale was considered high; 34–39 moderate; and less than 34 low [35]. This scale was selected because of its high reliability and validity; and it has previously been used in other South African studies [6–10]. Participants were considered to have burnout if they had high scores in the dimensions of emotional exhaustion or depersonalisation. The cut-off scores and the criteria for burnout used in this study are based on a 2016 systematic review, which identified the most widely used criteria to define burnout [35].

The Generalised Anxiety Disorder-7 (GAD-7) questionnaireis a 7-item questionnaire that is used as a screening tool for anxiety symptoms. A threshold score of > = 10 was used as it has a sensitivity of 89% and specificity of 82% to detect generalised anxiety disorder. It has been validated in the SA context [36].

The Patient Health Questionnaire-9 (PHQ-9) is a 9-item questionnaire that is used as a screening tool for depressive symptoms. A threshold score of > = 10 was used as it has a sensitivity and specificity of 88% for major depressive disorder. It has been validated for use in SA [37,38].

### Exposures

The study exposures explored were practitioner socio-demographic and organisational factors. Socio-demographic details collected were: age, gender, marital status, race, highest educational level, occupational rank and discipline. Data collected on the organisational factors included: number of overtime hours worked, support structures as well as causative and preventative factors pertaining to burnout. The following factors were rated as ‘very good’, ‘good’, ‘satisfactory’, ‘poor’ or ‘nil’: support by colleagues, clinical support by supervisor, hospital structure resources, impact on personal life, medical staffing at hospital, academic support and reimbursement. Participants selected the six most important factors that they perceived as contributing to burnout from 20 options provided, which included: working conditions (work load, number of work hours, working conditions, threat of disease, staff shortages, vacation limit, lack of equipment, tendency to overwork, physical safety); organisational factors (lack of management support, management problems, lack of supervision, public system-related frustration), and personal factors (low job satisfaction, lack of future opportunities, balancing work and personal life, regret of career choice, financial problems, insufficient training, substance use, business and insurance concerns). In addition, MDs chose the three most important factors that they believed prevented burnout from the following options: adequate staff, improved management/planning and support, mentorship, empathic administration, improved staff relationships, reduced hours, emotional support, acknowledgement and improved training.

### Statistical methods

Four analyses were conducted for this investigation, the first being descriptive statistics, to summarise the socio-demographic and occupational profile of participants. Second their clinical profiles (e.g. burnout, anxiety and depression) were assessed using descriptive statistics that were stratified by occupational rank, which was assessed using chi-square statistics (or Kruskal-Wallis for continuous variables). Third, we identified five major work environmental and occupational factors of burnout, with significant factors being identified using chi-square statistics. Lastly, we examined whether burnout is a significant covariate of anxiety and depression by fitting logistic regression models, which were adjusted by socio-demographic and occupational covariates. A significance of p<0.05 was used for statistical significance testing, with the data being analysed using Stata Version 15.

## Results

### Sociodemographic and occupational profile

At the five public sector hospitals, 329 participants were screened for eligibility. Seven were excluded as 5 declined and 2 were in part-time employ in the state sector. One hundred and seventy-two questionnaires were not returned, thus 150 MDs participated (response rate of 47%). Table 1 below summarises their socio-demographic and occupational profile. Their mean age was 32.6 years (SD = 7.0) with a range from 24 to 64 years.

**Table 1:** Socio-demographic and occupational profile in the ZABRE study on MDs (n = 150).

		N	%
Age category:	<30	54	36.0
	30–39	72	48.0
	40+	24	16.0
Gender:	Male	66	44.0
	Female	84	56.0
Marital status:	Single/Divorced	79	52.7
	Married	71	47.0
Race:	Black	29	19.3
	White/Coloured/Other	35	23.3
	Indian	86	57.3
Highest qualification:	MBChB or Diploma equivalent	78	52.0
	Specialist qualification—part I	36	24.0
	Specialist qualification—part II	36	24.0
Occupational rank	Intern	39	26.0
	Medical officer/clinical manager	55	36.7
	Registrar	25	16.7
	Specialist	31	20.7
Discipline:	General medicine	33	22.0
	Surgery	22	15.0
	Psychiatry	20	13.0
	Paediatrics	27	18.0
	Obstetrics and gynaecology	16	10.7
	Family medicine/trauma and emergency medicine	15	10.0
	Anaesthetics	17	11.3
Overtime (On and Off-site)	Combined hours	Mean = 91	SD = 46.25
		Median = 80	IQR = 22

The participants were mainly in the 30–39 year age group (n = 72, 48.0%); female (n = 84, 56.0%); and single/divorced (n = 79, 52.7%). Most were medical officers/clinical managers (36.7%), with 16.7% being registrars,16.7% specialists, and 26.0% interns.

### Burnout, anxiety and depression symptoms by occupational rank

Table 2 below describes the prevalence of burnout and its three subscales, as well as anxiety and depressive symptoms in relation to occupational rank. Burnout was reported by 88 (59.0%) of participants.

**Table 2:** Burnout, anxiety and depression by occupational rank in the ZABRE study on MDs.

		All		Intern (n = 39)		Medical Officers/Clinical Manager (n = 55)		Registrar (n = 25)		Specialist (n = 31)		χ^2^	df	P
		N	%	N	%	N	%	n	%	N	%			
Emotional exhaustion (EE):	High	73	48.7	24	61.5	22	40.0	15	60.0	12	38.7	8.69	6	0.19
	Moderate	32	21.3	7	17.9	14	25.5	2	8.0	9	29.0			
	Low	45	30.0	8	20.5	9	34.5	8	32.0	10	32.3			
Depersonalisation (DP):	High	68	45.3	27	69.2	17	30.9	11	44.0	13	29.0	22.8	6	**<0.01**
	Moderate	33	22	7	17.9	16	29.1	7	28.0	3	54.8			
	Low	49	32.7	5	12.8	22	40.0	7	28.0	15	16.1			
Burnout (High EE or High DP):	No	62	41.3	10	25.6	31	56.4	9	36.0	12	38.7	9.47	3	**0.02**
	Yes	88	58.7	29	74.4	24	43.6	16	64	19	61.3			
Personal accomplishment (PA):	High	33	22.0	2	5.1	16	29.1	6	24.0	9	29.0	14.6	6	**0.02**
	Moderate	52	34.7	6	41	23	41.8	6	24.0	7	22.6			
	Low	65	43.3	21	53.8	16	29.1	13	52.0	15	48.4			
GAD-7 (≥10):		Mdn = 5	IQR = 7	Mdn = 4	IQR = 6	Mdn = 5	IQR = 7	Mdn = 3	IQR = 6	Mdn = 5	IQR = 9	0.14	3	0.99
	No	120	80.0	32	82.1	44	80.0	22	88.0	22	71.0	2.68	3	0.44
	Yes	30	20.0	7	17.9	11	20.0	3	12.0	9	29.0			
PHQ-9 (≥10):		Mdn = 4	IQR = 7	Mdn = 4	IQR = 8	Mdn = 4	IQR = 8	Mdn = 3	IQR = 4	Mdn = 4	IQR = 9	0.82	3	0.85
	No	118	78.7	29	74.4	42	76.4	22	88.0	25	80.6	1.98	3	0.58
** **	Yes	32	21.3	10	25.6	13	23.6	3	12.0	6	19.4			

**Fig 1:**
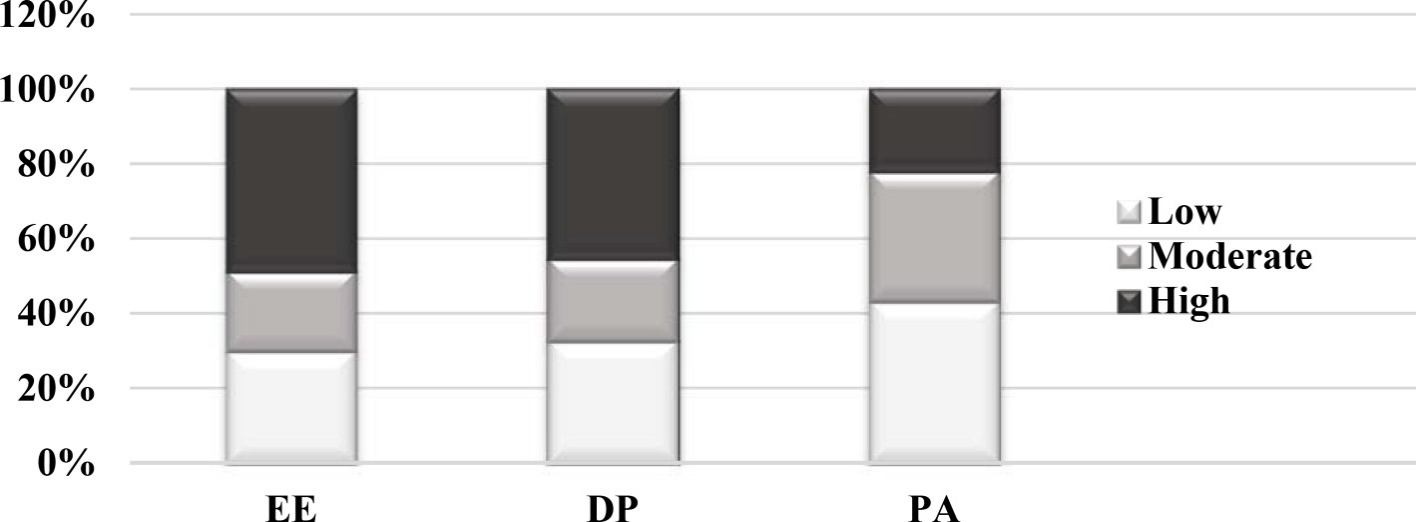
Dimensions of burnout for the ZABRE study on MDs.

Fig 1 below illustrates the MDs report of the three dimensions of burnout, with severity markers being categorised as low, moderate or severe.

The participants experienced significant levels of burnout in all dimensions: 73 (48.7%) having high levels of emotional exhaustion; 51 (34.0%) high levels of depersonalisation; and 55 (36.7%) low levels of personal accomplishment. The screening tests for depressive and anxiety symptoms were positive for 32 (21.3%) and 30 (20.0%) participants respectively. Burnout was associated with junior occupational rank (i.e. interns) (n = 28, p = 0.02), which was also associated with higher levels of depersonalisation (p<0.01) and lower levels of personal accomplishment (p = 0.02). No statistically significant associations were found between occupational rank and emotional exhaustion, anxiety and depressive symptoms.

### Work environmental and organisational factors associated with burnout

Table 3 describes the reported work environmental and organisational factors as experienced by the participants and their perceived association with burnout. Three factors associated with burnout were a lack of clinical support by supervisor (χ2 = 12.90; p<0.01); lack of hospital structure resources (χ2 = 14.56; p<0.01); and poor impact on personal life (χ2 = 19.61; p<0.01). The six most important factors reported by participants as being contributory to burnout are: staff shortages (75.0%), working conditions (71.0%), workload (63.0%), lack of equipment (53.0%), number of work hours (51.0%) and public system-related frustration (61.0%).

**Table 3:** Work environmental factors and burnout in the ZABRE study on MDs.

		All		Burnout				χ^2^	df	p
				No		Yes				
		n	%	n	%	n	%			
Rate support by colleagues:	Very good	35	23.6	17	27.4	18	20.9	2.04	3	0.56
	Good	56	37.8	25	40.3	31	36.0			
	Satisfactory	50	33.8	18	29.0	32	37.2			
	Poor	7	4.7	2	3.2	5	5.8			
Rate clinical support by supervisor:	Very good	27	18.6	17	27.9	10	11.9	12.90	3	**<0.01**
	Good	54	37.2	26	42.6	28	33.3			
	Satisfactory	48	33.1	16	26.2	32	38.1			
	Poor	16	11.0	2	3.3	14	16.7			
Rate hospital structure resources:	Good	6	4.2	5	8.3	1	1.2	14.56	2	**<0.01**
	Satisfactory	33	22.9	21	35.0	12	14.3			
	Poor	105	72.9	34	56.7	71	84.5			
Rate impact on personal life:	Very good	2	1.4	0	0.0	2	2.4	19.61	3	**<0.01**
	Good	9	6.4	8	14.3	1	1.2			
	Satisfactory	67	47.5	33	58.9	34	40.0			
	Poor	63	44.7	15	26.8	48	56.5			
Rate medical hospital staffing:	Good	6	4.0	2	3.2	4	4.6	6.47	3	0.09
	Satisfactory	44	29.5	25	40.3	19	21.8			
	Poor	98	65.8	35	56.5	63	72.4			
	None	1	0.7	0	0.0	1	1.1			
Rate academic support:	Very good	3	2.4	2	3.7	1	1.4	7.10	4	0.13
	Good	39	31.2	23	42.6	16	22.5			
	Satisfactory	46	36.8	17	31.5	29	40.8			
	Poor	34	27.2	11	20.4	23	32.4			
	None	3	2.4	1	1.9	2	2.8			
Rate reimbursement:	Very good	2	1.4	2	3.3	0	0.0	6.88	4	0.14
	Good	17	11.6	5	8.2	12	14.0			
	Satisfactory	60	40.8	30	49.2	30	34.9			
	Poor	43	29.3	16	26.2	27	31.4			
	None	25	17.0	8	13.1	17	19.8			
PBO (prevents burn out) improving recruitment:	Yes	81	54.0	33	53.2	48	54.5	0.03	1	0.87
	No	69	46.0	29	46.8	40	45.5			
PBO improved management:	Yes	90	60.0	34	54.8	56	63.6	1.17	1	0.28
	No	60	40.0	28	45.2	32	36.4			
PBO Support:	Yes	52	34.7	26	41.9	26	29.5	2.47	1	0.12
	No	98	65.3	36	58.1	62	70.5			
PBO mentorship:	Yes	19	12.7	7	11.3	12	13.6	0.18	1	0.67
	No	131	87.3	55	88.7	76	86.4			
PBO empathic administration:	Yes	22	14.7	8	12.9	14	15.9	0.26	1	0.61
	No	128	85.3	54	87.1	74	84.1			
PBO improved staff relationships:	Yes	35	23.3	17	27.4	18	20.5	0.99	1	0.32
	No	115	76.7	45	72.6	70	79.5			
PBO reduced hours:	Yes	74	49.3	25	40.3	49	55.7	3.43	1	0.06
	No	76	50.7	37	59.7	39	44.3			
PBO emotional support:	Yes	14	9.3	7	11.3	7	8.0	0.48	1	0.49
	No	136	90.7	55	88.7	81	92.0			
PBO acknowledgement:	Yes	29	19.3	14	22.6	15	17.0	0.71	1	0.40
	No	121	80.7	48	77.4	73	83.0			
PBO improved training:	Yes	26	17.3	12	19.4	14	15.9	0.30	1	0.58
	No	124	82.7	50	80.6	74	84.1			

### Regression analyses

Secondary analyses explored the relationships of anxiety and depressive symptoms to socio-demographic and occupational covariates as summarised in Table 4.

**Table 4:** Socio-demographic and occupational covariates of anxiety and depression based on regression models in the ZABRE study on MDs.

		GAD-7					PHQ-9				
		adj OR	SE	p	95% CI		adj OR	SE	p	95% CI	
Burnout (High EE or High DP):	[No]										
	Yes	8.62	5.98	**<0.01**	2.21	33.59	13.83	10.59	**<0.01**	3.08	62.00
Age category:	[<30]										
	30–39	29.54	43.9	**0.02**	1.61	543.69	6.33	6.35	0.07	0.88	45.28
	40+	27.62	46.18	**0.04**	1.04	732.21	21.23	27.75	**0.02**	1.64	275.2
Gender:	[Male]										
	Female	2.01	1.11	0.21	0.68	5.92	3.85	2.27	**0.02**	1.21	12.22
Marital status:	[Single/Divorced]										
	Married	0.46	0.29	0.22	0.13	1.59	0.42	0.27	0.18	0.12	1.49
Race:	[Black]										
	White/Coloured/Other	1.10	1.03	0.92	0.18	6.92	2.00	1.85	0.45	0.33	12.19
	Indian	1.97	1.69	0.43	0.37	10.59	2.55	2.16	0.27	0.48	13.43
Discipline:	[General medicine]										
	Surgery	0.26	0.30	0.25	0.03	2.62	0.57	0.54	0.55	0.09	3.60
	Psychiatry	0.19	0.23	0.16	0.02	1.95	0.13	0.15	0.09	0.01	1.34
	Paediatrics	0.77	0.61	0.74	0.16	3.63	0.84	0.69	0.83	0.17	4.24
	Obstetrics and gynaecology	2.57	2.28	0.29	0.45	14.66	4.41	3.98	0.1	0.75	25.85
	Family medicine/Trauma and emergency	0.35	0.44	0.4	0.03	4.14	0.55	0.59	0.58	0.07	4.48
	Anaesthetics	1.46	1.38	0.69	0.23	9.35	0.16	0.23	0.2	0.01	2.67
Occupation rank:	[Specialist]										
	Intern	9.20	15.17	0.18	0.36	233.18	11.06	14.93	0.08	0.79	155.66
	Medical officer band/Clinical Manager	1.04	0.76	0.96	0.25	4.33	6.45	5.76	**0.04**	1.12	37.09
	Registrar	0.37	0.37	0.32	0.05	2.60	1.04	1.14	0.97	0.12	8.93
Overtime (On and Off-site):	Combined hours	1.00	0.01	0.83	0.98	1.01	1.01	0.01	0.03	<0.01	1.02

Burnout was significantly associated with screening positive for anxiety (adjusted odds ratio [aOR] = 8.62, 95% CI 2.21–33.59; p<0.01) and depressive symptoms (aOR = 13.83, 95% CI 3.08–62.00; p<0.01) compared to those who were not burnt out. An increased risk of screening positive for anxiety symptoms was associated with the age groups of 30–39 years (aOR = 29.54, 95% CI 1.61–543.69; p = 0.02) and 40 years or older (aOR = 27.62, 95% CI 1.04–732.21; p = 0.04), compared to those younger than 30 years. In those 40 years or older, there was also an increased risk of screening positive for depressive symptoms (aOR = 21.23, 95% CI 1.64–275.2; p = 0.02) compared to those younger than 30 years. Depressive symptoms were also more likely in females (aOR = 3.85, 95% CI 1.21–12.22; p = 0.02) compared to males; and in the medical officer/clinical manager category compared to specialists (aOR = 6.45, 95% CI 1.12–37.09; p = 0.04).

## Discussion

The aim of this study was to investigate the prevalence of burnout, anxiety and depressive symptoms and their associations with practitioner (individual) and organisational factors among MDs employed at public sector hospitals in the eThekwini Municipality of KZN. The key results were that burnout, anxiety and depressive symptoms were common.Eighty-eight (59.0%) of the 150 participants experienced burnout, 30 (20%) screened positive for anxiety symptoms, and 32 (21.3%) for depressive symptoms. Burnout was associated with increased anxiety and depressive symptoms, with an association between burnout and individual and organisational factors.

While the prevalence of burnout in this study is consistent with global trends [39], it is lower than that reported in other SA studies [6–8]. Possible reasons for this may be that most of the previous studies were conducted in the WC [7,8], with only one national survey [6], and the core group in those studieswere junior doctors [7,8]. There may also be differences regarding the clinical settings in which these studies were conducted (resources); the race and sex distribution of MDs; sampling strategies and sample sizes [6–8].

The rates of anxiety and depressive symptoms reported in this study are aligned with international studies, although the figures vary based on the country, setting and tool administered [19–21]. The higher prevalence of depression uncovered in the WC (SA) studies may also be attributable to the participants mainly being junior doctors [7,30].

There does appear to be an association between burnout in relation to anxiety and depression. However, they are all considered distinct entities [33]. It has been difficult to ascertain the exact nature of these relationships, possibly due to the inconsistency in the burnout criteria used in the literature [2]. Due to a possible bi-directional association between burnout and negative affective states, a holistic approach needs to be considered to address burnout, anxiety and depressive symptoms relating to individual and organisational factors to improve MDs mental health.

Significant associations in this study that were aligned with global studies included the association between burnout and the following organisational factors: junior occupational rank (Maslach et al., 2001; West, Dyrbye, & Shanafelt, 2018); lack of support by clinical supervisor (Maslach et al., 2001); lack of hospital resources (West et al., 2018); and negative impact of work on personal life (Patel, Bachu, Adikey, Malik, & Shah, 2018). This suggests that junior doctors are very vulnerable, and that programs should focus on interns and provide more clinical supervision, mentorship and coping skills to assist these MDs.

Depersonalisation was significantly higher in interns in this study, however the 2013 WC study showed that depersonalisation affected community service medical officers (compulsory service in public sector for 1 year post internship) more than interns, medical officers, registrars and specialists [7]. This study was unable to recruit any community service medical officers (they are not based at academic hospitals in eThekwini); hence these findings are not comparable. Low levels of personal accomplishment were also significantly higher in interns, which is in keeping with the high burnout rates in this group. Staff shortages, working conditions, high workload, lack of equipment, long working hours and public system-related frustration are all factors that have been identified in previous studies as contributing to burnout [1,7,8,40–42]. Junior doctors are possibly more vulnerable due to their initial lack of capacity to manage these factors.

At an individual level, older age had an increased risk of screening positive for symptoms of anxiety. Age, female gender and occupational rank of medical officer/clinical manager increased the risk of screening positive for depression. Studies conducted internationally and within SA were inconclusive with findings relating to age and depression in MDs [20,21,30,43]. The finding of older age and increased anxiety and depressive symptoms may be due to the evolution of unrecognised burnout earlier on in the career of MDs and needs to be explored further in longitudinal studies. The finding of female gender and increased risk of depressive symptoms may be due to females being more empathic toward patients; prescribed gender roles; the necessity of females to navigate both their professional and domestic roles; and marginalisation by male colleagues [44–50].

### Limitations

The study was cross-sectional in nature hence causation could not be inferred. The instruments utilised rely on self-report measures, which may also introduce participant bias. While no validation studies have been done on the MBI-HSS in SA, the tool has been used in studies conducted in the WC and is considered the gold standard when assessing burnout. Generalisability of findings may have been hindered using convenience sampling and participants being selected only from training hospitals. Finally, the response rate of 47% was lower than those compared to other studies conducted in SA. This could reflect the differences in sampling strategies; a reluctance on the part of some participants who were possibly experiencing burnout to return the surveys; and difficulties in tracing MDs who had opted to return the surveys later. These factors could thus impact on the prevalence rates reported.

### Recommendations and conclusions

Burnout, anxiety and depressive symptoms are highly prevalent in MDs working in resource constrained KZN training hospitals. There appears to be an association between the syndrome of burnout and symptoms of anxiety and depression. In addition, younger MDs were more vulnerable to developing burnout, whilst older MDs were more likely to experience anxiety and depressive symptoms. Both individual-focused and organisational solutions [42,51] should be considered to mitigate the effect that staff shortages, working conditions, high workload, lack of equipment, long working hours and public system-related frustration have on the mental health of MDs working at these institutions, both from the perspectives of prevention and remediation.

As a priority, it is recommended that the Department of Health investigate the prevalence of burnout across different health facilities to understand the impact that its current practices are having on MDs. At the individual level, evidence-based strategies that can be used to reduce burnout, anxiety and depression in MDs include mindfulness, stress management and communication skills training, exercise programmes, self-care efforts and participation in small-group programmes oriented around promoting community, connectedness and meaning [42,51,52]. However, the prevention of burnout is preferable to putting the onus on the MDs to manage their stress and anxiety through medication or psychological interventions alone. Organisational strategies, although more difficult to address, should be focused on reducing work hours and workload, improving institutional support and advocacy for peer support with focus on junior doctors [42,51,52].

## Supporting information

S1 Fig Dimensions of burnout for the ZABRE study on MDs. (TIF)

S1 Table Socio-demographic and occupational profile in the ZABRE study on MDs (n = 150). (PDF)

S2 Table Burnout, anxiety and depression by occupational rank in the ZABRE study on MDs. (PDF)

S3 Table Work environmental factors and burnout in the ZABRE study on MDs. (PDF)

S4 Table Socio-demographic and occupational covariates of anxiety and depression based on regression models in the ZABRE study on MDs. (PDF)
