# Supplementary figures and images for: Burnout, anxiety and depression risk in medical doctors working in KwaZulu-Natal Province, South Africa: Evidence from a multi-site study of resource-constrained government hospitals in a generalised HIV epidemic setting
Source: PLoS One. 2020 Oct 14;15(10):e0239753. doi: 10.1371/journal.pone.0239753 (PMC7556533; doi:10.1371/journal.pone.0239753)

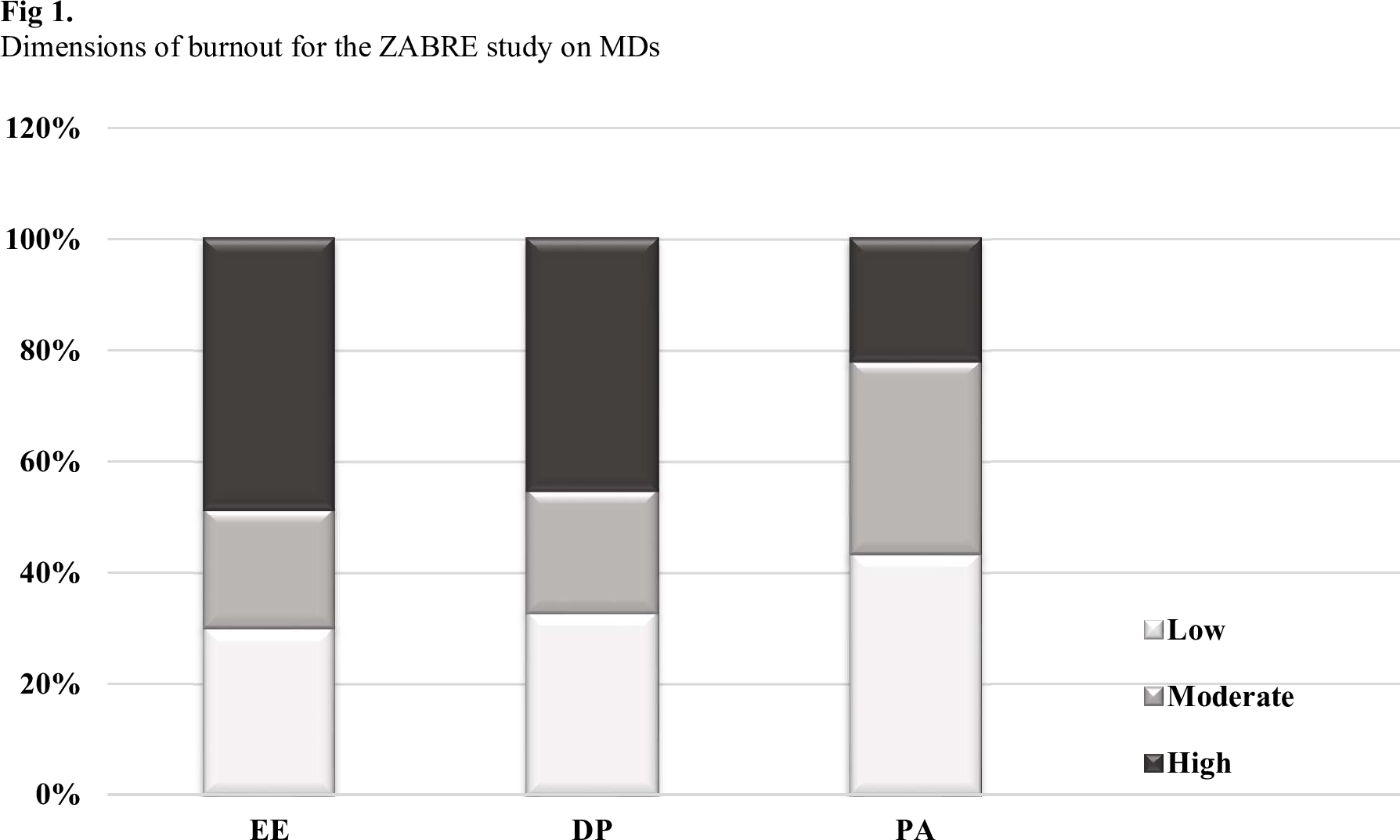

Supplement: S1 Fig — (TIF) [file pone.0239753.s001.tif]
