# Supplementary material for: Burnout, anxiety and depression risk in medical doctors working in KwaZulu-Natal Province, South Africa: Evidence from a multi-site study of resource-constrained government hospitals in a generalised HIV epidemic setting
Source: PLoS One. 2020 Oct 14;15(10):e0239753. doi: 10.1371/journal.pone.0239753 (PMC7556533; doi:10.1371/journal.pone.0239753)
Supplement: S1 Table — (PDF) [file pone.0239753.s002.pdf]

**Table 1.**
Socio-demographic and occupational profile in the ZABRE study on MDs (n=150)

| | | N | % |
|---|---|---|---|
| Age category: | <30 | 54 | 36.0 |
| | 30-39 | 72 | 48.0 |
| | 40+ | 24 | 16.0 |
| Gender: | Male | 66 | 44.0 |
| | Female | 84 | 56.0 |
| Marital status: | Single/Divorced | 79 | 52.7 |
| | Married | 71 | 47.0 |
| Race: | Black | 29 | 19.3 |
| | White/Coloured/Other | 35 | 23.3 |
| | Indian | 86 | 57.3 |
| Highest qualification: | MBChB or Diploma equivalent | 78 | 52.0 |
| | Specialist qualification - part I | 36 | 24.0 |
| | Specialist qualification - part II | 36 | 24.0 |
| Occupational rank | Intern | 39 | 26.0 |
| | Medical officer/clinical manager | 55 | 36.7 |
| | Registrar | 25 | 16.7 |
| | Specialist | 31 | 20.7 |
| Discipline: | General medicine | 33 | 22.0 |
| | Surgery | 22 | 15.0 |
| | Psychiatry | 20 | 13.0 |
| | Paediatrics | 27 | 18.0 |
| | Obstetrics and gynaecology | 16 | 10.7 |
| | Family medicine/trauma and emergency medicine | 15 | 10.0 |
| | Anaesthetics | 17 | 11.3 |
| Overtime (On and Off-site) | Combined hours | Mean = 91 | SD = 46.25 |
| | | Median = 80 | IQR =22 |
