# Supplementary material for: Burnout, anxiety and depression risk in medical doctors working in KwaZulu-Natal Province, South Africa: Evidence from a multi-site study of resource-constrained government hospitals in a generalised HIV epidemic setting
Source: PLoS One. 2020 Oct 14;15(10):e0239753. doi: 10.1371/journal.pone.0239753 (PMC7556533; doi:10.1371/journal.pone.0239753)
Supplement: S2 Table — (PDF) [file pone.0239753.s003.pdf]

Table 2.
Burnout, anxiety and depression by occupational rank in the ZABRE study on MDs

		All		Intern (n=39)		Medical Officers/Clinical Manager (n=55)		Registrar (n=25)		Specialist (n=31)		χ^2	df	P
		N	%	N	%	N	%	N	%	N	%			
Emotional exhaustion (EE):	High	73	48.7	24	61.5	22	40.0	15	60.0	12	38.7	8.69	6	0.19
	Moderate	32	21.3	7	17.9	14	25.5	2	8.0	9	29.0			
	Low	45	30.0	8	20.5	9	34.5	8	32.0	10	32.3			
Depersonalisation (DP):	High	68	45.3	27	69.2	17	30.9	11	44.0	13	29.0	22.8	6	**<0.01**
	Moderate	33	22	7	17.9	16	29.1	7	28.0	3	54.8			
	Low	49	32.7	5	12.8	22	40.0	7	28.0	15	16.1			
Burnout (High EE or High DP):	No	62	41.3	10	25.6	31	56.4	9	36.0	12	38.7	9.47	3	**0.02**
	Yes	88	58.7	29	74.4	24	43.6	16	64	19	61.3			
Personal accomplishment (PA):	High	33	22.0	2	5.1	16	29.1	6	24.0	9	29.0	14.6	6	**0.02**
	Moderate	52	34.7	6	41	23	41.8	6	24.0	7	22.6			
	Low	65	43.3	21	53.8	16	29.1	13	52.0	15	48.4			
GAD-7 (≥10):		Mdn = 5	IQR = 7	Mdn = 4	IQR = 6	Mdn = 5	IQR = 7	Mdn = 3	IQR = 6	Mdn = 5	IQR = 9	0.14	3	0.99
	No	120	80.0	32	82.1	44	80.0	22	88.0	22	71.0	2.68	3	0.44
	Yes	30	20.0	7	17.9	11	20.0	3	12.0	9	29.0			
PHQ-9 (≥10):		Mdn = 4	IQR = 7	Mdn = 4	IQR = 8	Mdn = 4	IQR = 8	Mdn = 3	IQR = 4	Mdn = 4	IQR = 9	0.82	3	0.85
	No	118	78.7	29	74.4	42	76.4	22	88.0	25	80.6	1.98	3	0.58
	Yes	32	21.3	10	25.6	13	23.6	3	12.0	6	19.4			
