# Supplementary material for: Burnout, anxiety and depression risk in medical doctors working in KwaZulu-Natal Province, South Africa: Evidence from a multi-site study of resource-constrained government hospitals in a generalised HIV epidemic setting
Source: PLoS One. 2020 Oct 14;15(10):e0239753. doi: 10.1371/journal.pone.0239753 (PMC7556533; doi:10.1371/journal.pone.0239753)
Supplement: S3 Table — (PDF) [file pone.0239753.s004.pdf]

Table 3.
Work environmental factors and burnout in the ZABRE study on MDs

		All		Burnout No		Yes		χ^2	df	p
		n	%	n	%	n	%			
Rate support by colleagues:	Very good	35	23.6	17	27.4	18	20.9	2.04	3	0.56
	Good	56	37.8	25	40.3	31	36.0			
	Satisfactory	50	33.8	18	29.0	32	37.2			
	Poor	7	4.7	2	3.2	5	5.8			
Rate clinical support by supervisor:	Very good	27	18.6	17	27.9	10	11.9	12.90	3	**<0.01**
	Good	54	37.2	26	42.6	28	33.3			
	Satisfactory	48	33.1	16	26.2	32	38.1			
	Poor	16	11.0	2	3.3	14	16.7			
Rate hospital structure resources:	Good	6	4.2	5	8.3	1	1.2	14.56	2	**<0.01**
	Satisfactory	33	22.9	21	35.0	12	14.3			
	Poor	105	72.9	34	56.7	71	84.5			
Rate impact on personal life:	Very good	2	1.4	0	0.0	2	2.4	19.61	3	**<0.01**
	Good	9	6.4	8	14.3	1	1.2			
	Satisfactory	67	47.5	33	58.9	34	40.0			
	Poor	63	44.7	15	26.8	48	56.5			
Rate medical hospital staffing:	Good	6	4.0	2	3.2	4	4.6	6.47	3	0.09
	Satisfactory	44	29.5	25	40.3	19	21.8			
	Poor	98	65.8	35	56.5	63	72.4			
	None	1	0.7	0	0.0	1	1.1			
Rate academic support:	Very good	3	2.4	2	3.7	1	1.4	7.10	4	0.13
	Good	39	31.2	23	42.6	16	22.5			
	Satisfactory	46	36.8	17	31.5	29	40.8			
	Poor	34	27.2	11	20.4	23	32.4			
	None	3	2.4	1	1.9	2	2.8			
Rate reimbursement:	Very good	2	1.4	2	3.3	0	0.0	6.88	4	0.14
	Good	17	11.6	5	8.2	12	14.0			
	Satisfactory	60	40.8	30	49.2	30	34.9			
	Poor	43	29.3	16	26.2	27	31.4			
	None	25	17.0	8	13.1	17	19.8			

Table 3. (continued)
Work environmental factors and burnout in the ZABRE study on MDs

		All		Burnout No		Yes		χ^2	df	p
		n	%	n	%	n	%			
PBO (prevents burn out) improving recruitment:	Yes	81	54.0	33	53.2	48	54.5	0.03	1	0.87
	No	69	46.0	29	46.8	40	45.5			
PBO improved management:	Yes	90	60.0	34	54.8	56	63.6	1.17	1	0.28
	No	60	40.0	28	45.2	32	36.4			
PBO Support:	Yes	52	34.7	26	41.9	26	29.5	2.47	1	0.12
	No	98	65.3	36	58.1	62	70.5			
PBO mentorship:	Yes	19	12.7	7	11.3	12	13.6	0.18	1	0.67
	No	131	87.3	55	88.7	76	86.4			
PBO empathic administration:	Yes	22	14.7	8	12.9	14	15.9	0.26	1	0.61
	No	128	85.3	54	87.1	74	84.1			
PBO improved staff relationships:	Yes	35	23.3	17	27.4	18	20.5	0.99	1	0.32
	No	115	76.7	45	72.6	70	79.5			
PBO reduced hours:	Yes	74	49.3	25	40.3	49	55.7	3.43	1	0.06
	No	76	50.7	37	59.7	39	44.3			
PBO emotional support:	Yes	14	9.3	7	11.3	7	8.0	0.48	1	0.49
	No	136	90.7	55	88.7	81	92.0			
PBO acknowledgement:	Yes	29	19.3	14	22.6	15	17.0	0.71	1	0.40
	No	121	80.7	48	77.4	73	83.0			
PBO improved training:	Yes	26	17.3	12	19.4	14	15.9	0.30	1	0.58
	No	124	82.7	50	80.6	74	84.1			
