# Supplementary material for: Burnout, anxiety and depression risk in medical doctors working in KwaZulu-Natal Province, South Africa: Evidence from a multi-site study of resource-constrained government hospitals in a generalised HIV epidemic setting
Source: PLoS One. 2020 Oct 14;15(10):e0239753. doi: 10.1371/journal.pone.0239753 (PMC7556533; doi:10.1371/journal.pone.0239753)
Supplement: S4 Table — (PDF) [file pone.0239753.s005.pdf]

Table 4.

Sociodemographic and occupational covariates of anxiety and depression based on regression models in the ZABRE study on MDs

		GAD-7					PHQ-9				
		adj OR	SE	p	95% CI		adj OR	SE	p	95% CI	
Burnout (High EE or High DP):	[No]										
	Yes	8.62	5.98	**<0.01**	2.21	33.59	13.83	10.59	**<0.01**	3.08	62.00
Age category:	[<30]										
	30-39	29.54	43.9	**0.02**	1.61	543.69	6.33	6.35	0.07	0.88	45.28
	40+	27.62	46.18	**0.04**	1.04	732.21	21.23	27.75	**0.02**	1.64	275.2
Gender:	[Male]										
	Female	2.01	1.11	0.21	0.68	5.92	3.85	2.27	**0.02**	1.21	12.22
Marital status:	[Single/Divorced]										
	Married	0.46	0.29	0.22	0.13	1.59	0.42	0.27	0.18	0.12	1.49
Race:	[Black]										
	White/Coloured/Other	1.10	1.03	0.92	0.18	6.92	2.00	1.85	0.45	0.33	12.19
	Indian	1.97	1.69	0.43	0.37	10.59	2.55	2.16	0.27	0.48	13.43
Discipline:	[General medicine]										
	Surgery	0.26	0.30	0.25	0.03	2.62	0.57	0.54	0.55	0.09	3.60
	Psychiatry	0.19	0.23	0.16	0.02	1.95	0.13	0.15	0.09	0.01	1.34
	Paediatrics	0.77	0.61	0.74	0.16	3.63	0.84	0.69	0.83	0.17	4.24
	Obstetrics and gynaecology	2.57	2.28	0.29	0.45	14.66	4.41	3.98	0.1	0.75	25.85
	Family medicine/Trauma and emergency	0.35	0.44	0.4	0.03	4.14	0.55	0.59	0.58	0.07	4.48
	Anaesthetics	1.46	1.38	0.69	0.23	9.35	0.16	0.23	0.2	0.01	2.67
Occupation rank:	[Specialist]										
	Intern	9.20	15.17	0.18	0.36	233.18	11.06	14.93	0.08	0.79	155.66
	Medical officer band/Clinical Manager	1.04	0.76	0.96	0.25	4.33	6.45	5.76	**0.04**	1.12	37.09
	Registrar	0.37	0.37	0.32	0.05	2.60	1.04	1.14	0.97	0.12	8.93
Overtime (On and Off-site):	Combined hours	1.00	0.01	0.83	0.98	1.01	1.01	0.01	0.03	<0.01	1.02
